# Simultaneous real-time visible and infrared video with single-pixel detectors

**DOI:** 10.1038/srep10669

**Published:** 2015-05-22

**Authors:** Matthew. P. Edgar, Graham M. Gibson, Richard W. Bowman, Baoqing Sun, Neal Radwell, Kevin J. Mitchell, Stephen S. Welsh, Miles J. Padgett

**Affiliations:** 1 SUPA, School of Physics and Astronomy, University of Glasgow, Glasgow, G12 8QQ, UK; 2 Department of Physics, Cavendish Laboratory, University of Cambridge, Cambridge CB3 0HE, UK

## Abstract

Conventional cameras rely upon a pixelated sensor to provide spatial resolution. An alternative approach replaces the sensor with a pixelated transmission mask encoded with a series of binary patterns. Combining knowledge of the series of patterns and the associated filtered intensities, measured by single-pixel detectors, allows an image to be deduced through data inversion. In this work we extend the concept of a ‘single-pixel camera’ to provide continuous real-time video at 10 Hz , simultaneously in the visible and short-wave infrared, using an efficient computer algorithm. We demonstrate our camera for imaging through smoke, through a tinted screen, whilst performing compressive sampling and recovering high-resolution detail by arbitrarily controlling the pixel-binning of the masks. We anticipate real-time single-pixel video cameras to have considerable importance where pixelated sensors are limited, allowing for low-cost, non-visible imaging systems in applications such as night-vision, gas sensing and medical diagnostics.

When multi-pixel sensors are not available due to cost or technological constraints, imaging systems require alternative approaches. A number of related approaches use spatially structured illumination^1^,^2^,^3^ or structured detection^4^,^5^ and a single-pixel detector to deduce an image. Perhaps the most obvious of these approaches is to raster scan a spatially selective detector over the field of view and rely upon the temporal analysis of the back-scattered light to give the intensity of every pixel in the image. Although giving a complete image, clearly this approach has an optical efficiency that scales inversely with the number of pixels in the image.

Instead of scanning a single detector over the whole image an alternative is to measure many pixels simultaneously, which in practice improves the measurement signal-to-noise. Broadly-termed aperture coding, one implementation scheme uses a series of binary transmission masks applied using a spatial light modulator to the image formed by a lens, and a single detector to measure the transmitted intensity. The known series of mask patterns and the measured intensities can be combined and inverted, using a variety of algorithms, to give a good estimate of the image. The use of a single detector to obtain the image data leads this technique to be called a ‘single-pixel camera’^4^,^6^. We note at this point that the single-pixel camera has much in common with the field of computational ghost imaging^7^,^8^,^9^,^10^,^11^, whereupon the latter uses knowledge of projected illumination patterns and measured back-scattered signals. Both single-pixel cameras and computational ghost imaging systems use similar algorithms applied to the back scattered intensities to deduce the image.

For both aforementioned approaches utilising single-pixel detectors to reconstruct images there exists a trade-off between acquisition time and image resolution, which results from the finite display rate of the spatial light modulator. Importantly however, most natural images exhibit similar characteristics, for example sparsity in their spatial frequencies, which allow for compressive techniques to represent images with less information. Indeed the field of compressed sensing asserts that an image can be recovered with far fewer measurements than the Nyquist limit. Recently, there has been considerable interest in the development of advanced compressive algorithms for the acquisition of video^12^,^13^,^14^,^15^,^16^,^17^,^18^,^19^,^20^. However impressive the compression rates of these approaches, the associated reconstruction times greatly exceed the acquisition time thereby prohibiting use in real-time video systems for human interfacing.

## Results

In this work we demonstrate real-time video from a single-pixel camera for visible and short-wave infrared (SWIR) wavelengths without the requirement for lengthy post-processing. Our system utilises a high-speed digital micro-mirror device (DMD) to apply spatial masks from which the transmitted light is spectrally filtered simultaneously onto four separate single-pixel detectors, corresponding to the red, green, blue and SWIR colour channels. We note that this experimental system has a similar configuration to that in Ref [4, 6], with the addition of simultaneous spectral filtering. However, key to this approach is the implementation of an efficient iterative computer algorithm that stores only the current frame in memory and thus can operate continuously, which is an important feature for this technology in applications. We compare real-time colour video (red, green, blue) and SWIR video (800-1800 nm) acquired and processed at frame rates of ~10 Hz with a resolution of 32 × 32 pixels or ~2.5 Hz at a resolution of 64 × 64 pixels. Furthermore, we demonstrate the use of real-time image optimisation similar to techniques used in existing ‘compressed sensing’ work^4^,^21^,^22^, in the presence of moderate and excessive noise. In addition, we demonstrate the recovery of high-resolution detail in real-time by arbitrarily modifying both the DMD region of interest and mirror binning.

Our modified single-pixel camera demonstrates simultaneous acquisition of multispectral images in the visible and shortwave infrared. Obtaining colour and SWIR images provide an intuitive demonstration of the technology, showcasing the perfect pixel registration inherent with this approach. However, in principle these types of systems can be extended for imaging at mid-infrared and terahertz wavelengths, where existing detector arrays are very expensive or have inherent limitations, such as microbolometer arrays which require cooling for improved sensitivity or low-resolution Schottky diode arrays that require scanning.

**Figure 1:**
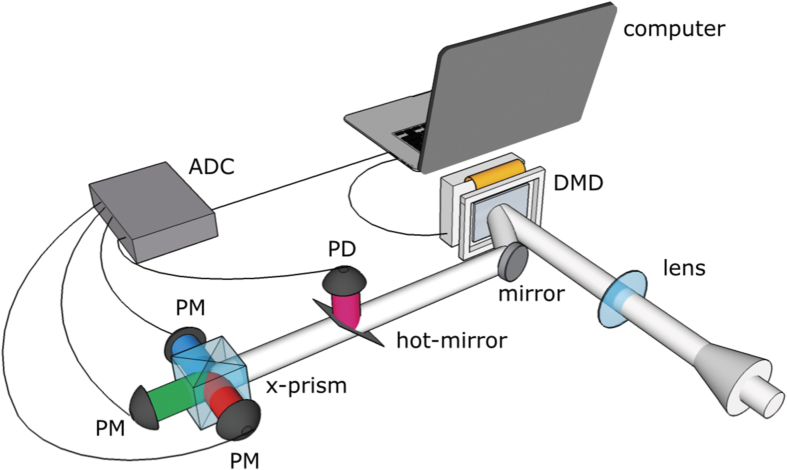
Experimental setup for real-time video with single-pixel detectors. A lens is used to form an image of the scene onto the digital-micromirror-device (DMD). The spatially filtered light is directed onto a hot-mirror, which reflects SWIR light onto an InGaAs photodetector (PD) and transmits visible light. A dichroic prism (x-prism) subsequently filters the visible light into red, green and blue output ports, sensed by three identical photomultipliers (PM’s). The measured intensities are digitised by an analog-to-digital converter (ADC) for computer processing.

The optical design concept is based upon a high-speed digital light projector for which the light source is replaced with a detection system incorporating a hot mirror to separate SWIR and visible light. Subsequently, a dichroic prism is used to separate visible light into red, green and blue spectral bands. Photomultipliers (PMT’s) are used to sense the visible colour channels and an InGaAs photodiode is used to sense the SWIR light (see Fig. 1). The controller board for the DMD allows binary patterns to be preloaded and then displayed at a maximum rate of 20.7 kHz. Each mirror on the DMD can be electronically actuated to one of two states representing ‘on’ or ‘off’ (transmissive or opaque)^23^. For every pattern displayed, the controller board also provides an output synchronisation TTL signal that is used to trigger signal acquisition on a high-dynamic range, analogue-to-digital converter (ADC), capable of acquiring 250 k samples/s.

As with any imaging system, the image quality is dependent upon the level of noise on the detector, whether it be a single-pixel or a sensor array. With single-pixel imaging technology, image quality can be optimised via both optical and computational techniques. Similar to the work of Ref [4,6], we perform optical optimisation by making use of PMT’s as the single-pixel detectors for detection of visible light, which enables the system to operate under low-light conditions. In this experiment we make use of the well-known Hadamard matrices^24^, which are binary matrices that form a complete orthonormal set and where each pattern contains an equal number of 1’s and −1’s, representing ‘on’ and ‘off’ respectively for each mask applied to the DMD. Each row from the Hadamard matrix, when reshaped to 2D, contains a different subset of spatial frequencies, such that an 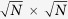 pixel image can be fully sampled with *N* Hadamard patterns. However, to preserve orthogonality, this approach relies on sensing the light reflected in both outputs of the DMD, or alternatively, by displaying each pattern and immediately succeeding it by its inverse. The former requires the use of two detectors for each waveband, while the latter approach used here halves the maximum achievable frame rate. Both approaches utilise the difference between the two measurements to provide a zero-mean differential signal^25^, which is similar to lock-in detection at 22 kHz and 11 kHz respectively, helping to eliminate unwanted sources of noise.

Given a sequence of *N* orthonormal pattern pairs *A*_*i*,*j*_, where the pixel values are assigned ±1 (*i* is the pixel number and *j* is the pattern number), the corresponding differential signals are *S*_*j*,*n*_. For a pattern sequence the *n*^*th*^ image estimate of thebject *O*_*x*,*y*,*n*_, reformatted as a column vector of pixel values, *O*_*i*,*n*_, can be estimated simply as
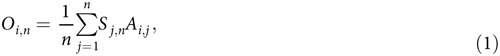
which for *n* = *N* provides perfect reconstruction of the image in the absence of noise and is equivalent to the least-squares solution to the data. However, most images, *O*_*x*,*y*_, are sparse, not in their intensity values but in their spatial frequencies. The field of compressed sensing takes advantage of this fact by using only a subset of measurement patterns 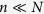, whilst still achieving a good estimate of the image. However, depending on the level of compression, most implementations require considerable processing time and therefore these techniques do not lend themselves well to real-time single-pixel video systems.

In this work we show that by employing relatively low-resolution Hadamard matrices, an image can be sampled and iteratively reconstructed in real-time from Eq. (1) at up to 10 Hz. However, Eq. (1) can still yield poor quality reconstructions under certain conditions, for example when the measurement SNR is low or when performing compressive sampling. Therefore an optimisation algorithm can instead be employed which uses the output of Eq. (1) and the measured signals and patterns within a forward-model. As an alternative to optimising for sparsity of the spatial frequencies, we perform minimisation of the image spatial-curvature, the latter being far quicker to compute and hence more applicable to real-time video. In our system optimisation is applied to each of the colour plane images separately, based on minimisation of its spatial-curvature and its frame-to-frame temporal derivative. The *n*^*th*^ image of the sequence is obtained by minimisation of its cost function, *C*_*n*_, given by
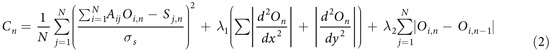
where *σ*_*s*_ is the standard deviation of the noise associated with the measurement of *S*_*j*,*n*_ and *O*_*n*_ is the image expressed in 2D form. The rst term of Eq. (2) corresponds to a minimisation of *χ*^2^/*N* of the image with respect to the measured data; the second term represents a minimisation of the total spatial-curvature and the third term corresponds to a minimisation of the difference between the current and previous image. Values of λ_1_ and λ_2_ are picked empirically to ensure that, once optimised, 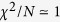 Utilising an image resolution of 32 × 32 pixels, the optimisation algorithm runs approximately 5× faster than the total time for displaying 1024 pattern pairs, allowing suitable time for convergence before a new set of signals is supplied to the algorithm for the following frame.

**Figure 2:**
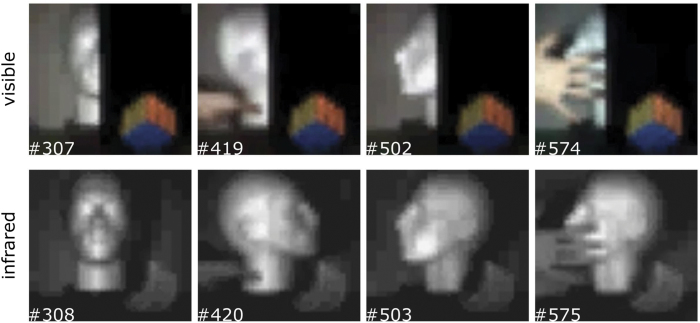
Sample of video frames reconstructed in real-time for visible and SWIR wavelengths simultaneously. The top and bottom rows respectively show video frames for full-colour (red,green,blue) and SWIR (800 − 1800 nm). Both reconstructions have been fully sampled at 32 × 32 pixel resolution and up-sampled and interpolated to 64 × 64 pixels in real-time with no additional time-lag.

**Figure 3:**
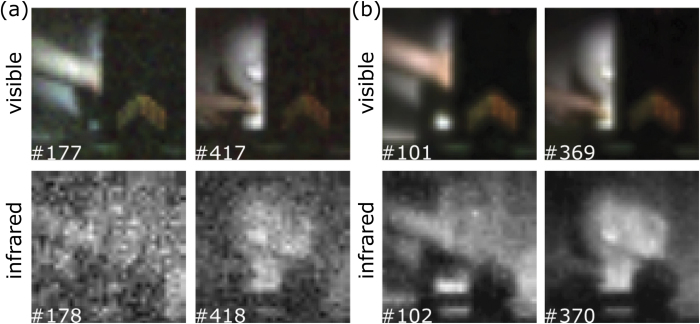
Sample of video frames reconstructed in low light conditions for visible and SWIR wavelengths simultaneously, whilst utilising an iterative and optimisation algorithm. The top and bottom rows respectively show video frames for full-colour (red,green,blue) and SWIR (800–1800 nm). Frames reconstructed using the iterative algorithm (**a**) show uniform noise across the image compared to frames reconstructed using the optimisation algorithm (**b**) at the expense of image resolution. Both reconstructions have been fully sampled at 32 × 32 pixel resolution and up-sampled and interpolated to 64 × 64 pixels in real-time with no additional time-lag.

As the theoretical framework supporting the iterative and optimisation algorithm have been outlined in Eqs. (1) and (2) respectively, we herein provide experimental reconstructions when operating the system in different conditions. Four frames extracted from a video (see supplementary material: Video 1) are shown in Fig. 2, obtained when operating the camera in good lighting conditions. Each *N* pixel frame is obtained by displaying *N* orthogonal pattern pairs and iteratively reconstructing using Eq. (1). The top row of Fig. 2 shows that good quality full-colour real-time video is acquired and we observe that no visible light penetrates the infrared-transmitting filter located at the right-hand side of each frame. The bottom row of Fig. 2 shows the corresponding SWIR video frames. As expected the entire scene located behind the infrared filter is revealed by measuring light on the the InGaAs detector. In addition, we demonstrate the system performance under low-light conditions, by attenuating the light received at the lens using an ND filter. The frames shown in Fig. 3(a,b) have been reconstructed using Eqs. (1) and Eq. (2), respectively. We observe a noise reduction within the image at the expense of spatial resolution as expected, when using optimisation parameters λ_1_ = 3 × 10^−5^ and λ_2_ = 0.75.

In addition to reconstructing fully sampled real-time video at a frame rate of ~10 Hz for an image resolution of 32 × 32 pixels, increased image resolutions are also possible. At increased resolutions however, obtaining real-time video demands efficient compressed sensing techniques to achieve similar frame-rates. In choosing the optimal compressed sensing approach we note that typical images can be represented by a subset of Hadamard patterns. Hence by utilising only the patterns whose measured signals yield ‘significant’ magnitudes, a form of compressed sensing is applied. In other words, we can choose to display only a subset of the complete pattern library (stored on the controller board memory), which are predicted to give the highest signals in the subsequent frame. This compression technique works well for static scenes, since the measured signals from the previous frame can be used to inform the choice of patterns in the following frame. However this approach is less robust for monitoring dynamic changes within a scene in real-time as the ‘significant’ patterns for the next frame can change. Therefore to allow the ‘significant’ subset of patterns to continuously adapt to a dynamic scene, a small random subset from the remaining pattern library can be utilised. We term this iterative compressed sensing approach an ‘evolutionary’ compressive technique^26^.

**Figure 4:**
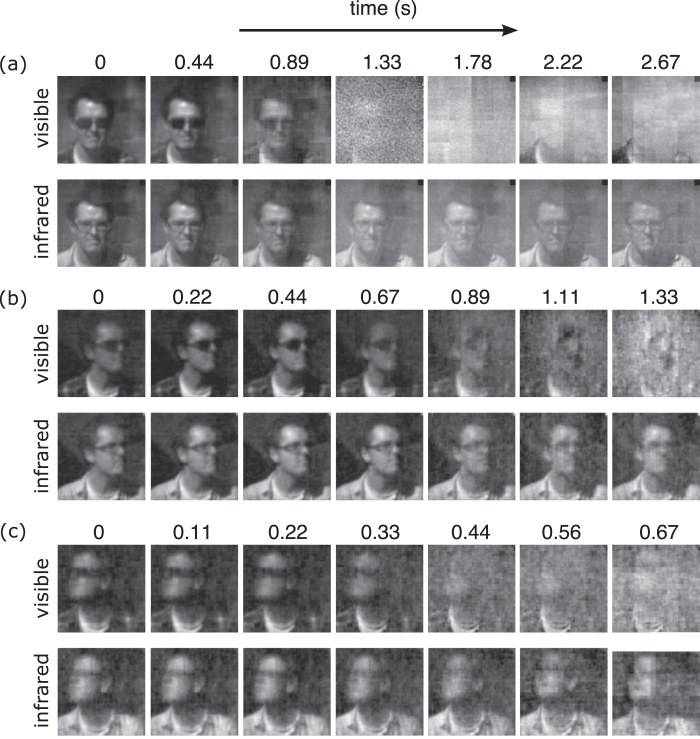
Sample of video frames reconstructed in the presence of smoke for different levels of compressed sensing. The frames from each video are reconstructed at 64 × 64 pixel resolution and up-sampled and interpolated to 128 × 128 pixels in real-time with no additional time-lag. The top and bottom rows show a sample of video frames for visible (red,green,blue) and SWIR (800–1800 nm), when sampled at (**a**) 100%, (**b**) 50% and (**c**) 25% compression. An optimisation algorithm is employed for compressively sensed frames as shown in (**b**) and (**c**).

In a separate experiment the system was modified by removing the dichroic prism and two photomultipliers such that a single photomultiplier was used to measure visible light. A comparison was then made between visible and SWIR channels whilst operating outdoors in daylight, while a commercial smoke machine was used to fill the scene. Fig. 4(a) shows a sample of visible and infrared frames reconstructed at 64 × 64 pixel resolution using Eq. (1) at a frame rate of ~2.5 Hz, and continuously using 4096 Hadamard pattern pairs, equating to no compression (see supplementary material: Video 2). We observe that the subject’s tinted sunglasses become transparent at SWIR compared to visible wavelengths. Moreover, the presence of smoke is shown to overwhelm the visible channel due to scattering, while the subject remains clear in the SWIR channel. In Fig. 4(b,c) the number of pattern pairs continuously displayed on the DMD was 2048 and 1024 respectively, equivalent to 50% and 25% compression, providing frame rates of ~5 Hz and ~10 Hz, respectively (see supplementary material: Video 3 and Video 4 respectively). Reconstructions were made utilising Eq. (2) with λ_1_ = 1 × 10^−4^ and λ_2_ = 0.8.

It is clear that due to the fixed frame-rate of the DMD, a trade-off exists between the frame-rate and pixel number with single-pixel camera architectures. One technique described in Ref. [15] utilises multi-scale sampling resolution in order to permit reconstruction of low-resolution frames throughout the acquisition process. By utilising the low-resolution images, an estimate for the motion in the scene is calculated, and later used as an additional constraint for compressive reconstruction of higher-resolution video frames, providing improved robustness to temporal blur. However, the requirement for post-processing with this approach still prevents real-time high-resolution video.

**Figure 5:**
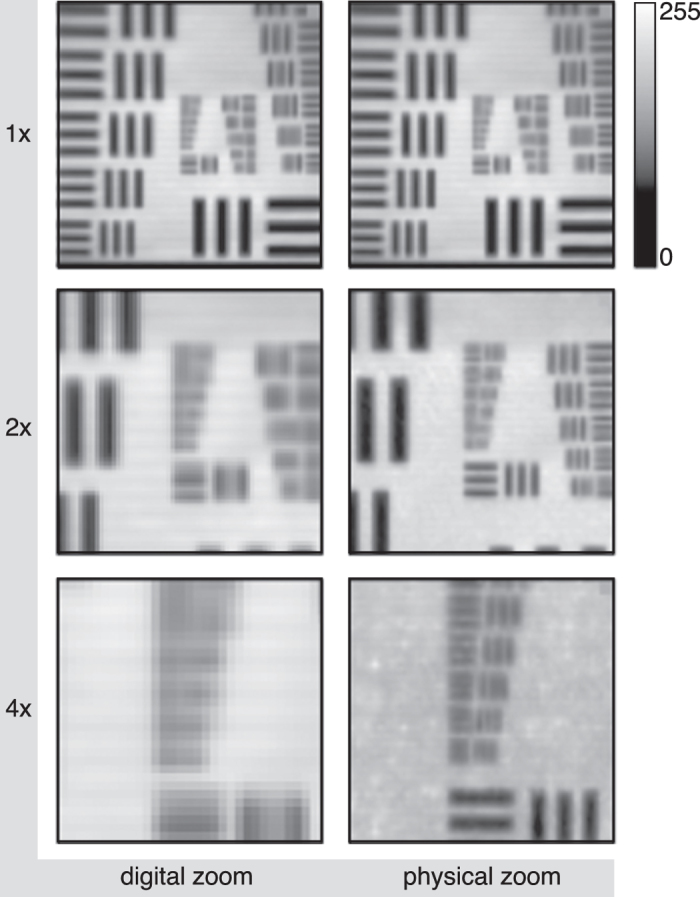
Sample video frames acquired for different levels of zoom. The object, a USAF test target, is located at a distance of 2 meters from the camera. The left column shows the image when sampled fully at 64 × 64 pixel resolution with fixed mirror binning of 12 × 12 and digitally zooming into the central region of interest by a factor of 2 and 4. In contrast the right column shows the image fully sampled at 64 × 64 pixel resolution, but for 12 × 12, 6 × 6 and 3 × 3 mirror binning, equivalent to providing a physical zoom of 1×, 2× and 4×, respectively.

We propose a compromise in order to achieve increased spatial-resolution, maintain high frame-rates and is relatively simple to employ, which is to sample over an arbitrarily defined region of interest but maintain the orthogonal sampling matrix dimensions. This can be achieved in practice by changing the mirror binning, whereby ‘unused’ mirrors remain in the ‘off’ state and are hence not sensed by the photodetector. By reducing the mirror binning, the angular resolution is increased, equivalent to an optical zoom. However, importantly no optical component is moved with this approach, instead for every additional level of zoom, an extra pattern library is preloaded into the onboard memory and selected for display on the DMD. Fig. 5 shows the result of 12 × 12, 6 × 6 and 3 × 3 mirror binning, which is analogous to providing 1×, 2× and 4× physical zoom of the image for a predetermined resolution of 64 × 64 pixels.

Nevertheless, the challenge of obtaining full-frame, high-resolution video in real-time from single-pixel detectors remains. Perhaps this will be made possible in the future with improved computer processing performance and advanced video-based compressed sensing techniques or through efficient optical and computational multiplexing^27^.

The data used to produce the content of this manuscript is available at: http://dx.doi.org/10.5525/gla.researchdata.167

## Discussion

We have demonstrated a real-time video system utilising single-pixel detectors for imaging simultaneously at visible and short-wave infrared wavelengths. The maximum frame rate of our camera is ~10 H*z*. We have demonstrated the infrared real-time imaging system when monitoring a variety of scenes containing an infrared filter, sunglasses, smoke and under different lighting conditions, similar in character to results reported with conventional SWIR detector arrays. Once matured this technology could lead to significantly cheaper SWIR imaging devices. When operating in low-light conditions, or performing compressive sampling, an efficient optimisation algorithm was shown to considerably improve the image quality. In order to increase spatial resolution of images whilst maintaining high-frame rates we have shown a simple approach for arbitrarily modifying the mirror binning and utilising a specific region of interest on the DMD. In practice this region of interest may be automated or arbitrarily defined by the camera operator. We anticipate that the large operational bandwidth afforded by DMD technology will enable real-time imaging from such single-pixel camera systems to have widespread application in industry, medicine and defence, at wavelengths were detector arrays are limited or costly.

## Additional Information

**How to cite this article**: Edgar, M. P. *et al.* Simultaneous real-time visible and infrared video with single-pixel detectors. *Sci. Rep.* **5**, 10669; doi: 10.1038/srep10669 (2015).

## Supplementary Material

Supporting Information

Supporting Information

Supporting Information

Supporting Information

Supporting Information
