# Supplementary material for: Simultaneous real-time visible and infrared video with single-pixel detectors
Source: Sci Rep. 2015 May 22;5:10669. doi: 10.1038/srep10669 (PMC4650679; doi:10.1038/srep10669)
Supplement: Supporting Information [file srep10669-s1.pdf]

Supplementary Information for

Simultaneous real-time visible and infrared video with single-pixel detectors

Matthew P. Edgar*, Graham M. Gibson, Richard W. Bowman, Baoqing Sun,

Neal Radwell, Kevin M. Mitchell, Stephen S. Welsh & Miles J. Padgett

Correspondence to: matthew.edgar@glasgow.ac.uk

This Supplementary Information includes:

Captions for videos 1-4

Captions for videos 1-4

Video 1: Video reconstructed in real-time for visible and SWIR wavelengths simultaneously. The visible band is displayed in full-colour (red,green,blue) and SWIR (800 - 1800 nm). Both reconstructions have been fully sampled at 32 x 32 pixel resolution and up-sampled and interpolated to 64 x 64 pixels in real-time with no additional time-lag.

Video 2: Video reconstructed in the presence of smoke when operating the camera with 100% sampling, i.e. no compression using the iterative algorithm described in the text. The visible and shortwave infrared video is reconstructed at 64 × 64 pixel resolution and up-sampled and interpolated to 128 × 128 pixels in real-time with no additional time-lag.

Video 3: Video reconstructed in the presence of smoke when operating the camera with 50% sampling, using the optimisation algorithm and evolutionary pattern selection described in the text. The visible and shortwave infrared video is reconstructed at 64 × 64 pixel resolution and up-sampled and interpolated to 128 × 128 pixels in real-time with no additional time-lag.

Video 4: Video reconstructed in the presence of smoke when operating the camera with 25% sampling, using the optimisation algorithm and evolutionary pattern selection described in the text. The visible and shortwave infrared video is reconstructed at 64 × 64 pixel resolution and up-sampled and interpolated to 128 × 128 pixels in real-time with no additional time-lag.
